# Goodness-of-fit two-phase sampling designs for time-to-event outcomes: a simulation study based on New York University Women’s Health Study for breast cancer

**DOI:** 10.1186/s12874-023-01950-4

**Published:** 2023-05-19

**Authors:** Myeonggyun Lee, Jinbo Chen, Anne Zeleniuch-Jacquotte, Mengling Liu

**Affiliations:** 1 Department of Population Health, New York University Grossman School of Medicine, New York, NY 10016 USA; 2 Department of Biostatistics, Epidemiology and Informatics, Perelman School of Medicine, University of Pennsylvania, Philadelphia, PA 19104 USA; 3 Department of Environmental Medicine, New York University Grossman School of Medicine, New York, NY 10016 USA

**Keywords:** Case-cohort, Cox proportional hazards model, Hazard ratio estimation, Inverse probability weighting, Relative efficiency

## Abstract

**Background:**

Sub-cohort sampling designs such as a case-cohort study play a key role in studying biomarker-disease associations due to their cost effectiveness. Time-to-event outcome is often the focus in cohort studies, and the research goal is to assess the association between the event risk and risk factors. In this paper, we propose a novel goodness-of-fit two-phase sampling design for time-to-event outcomes when some covariates (e.g., biomarkers) can only be measured on a subgroup of study subjects.

**Methods:**

Assuming that an external model, which can be the well-established risk models such as the Gail model for breast cancer, Gleason score for prostate cancer, and Framingham risk models for heart diseases, or built from preliminary data, is available to relate the outcome and complete covariates, we propose to oversample subjects with worse goodness-of-fit (GOF) based on an external survival model and time-to-event. With the cases and controls sampled using the GOF two-phase design, the inverse sampling probability weighting method is used to estimate the log hazard ratio of both incomplete and complete covariates. We conducted extensive simulations to evaluate the efficiency gain of our proposed GOF two-phase sampling designs over case-cohort study designs.

**Results:**

Through extensive simulations based on a dataset from the New York University Women’s Health Study, we showed that the proposed GOF two-phase sampling designs were unbiased and generally had higher efficiency compared to the standard case-cohort study designs.

**Conclusion:**

In cohort studies with rare outcomes, an important design question is how to select informative subjects to reduce sampling costs while maintaining statistical efficiency. Our proposed goodness-of-fit two-phase design provides efficient alternatives to standard case-cohort designs for assessing the association between time-to-event outcome and risk factors. This method is conveniently implemented in standard software.

**Supplementary Information:**

The online version contains supplementary material available at 10.1186/s12874-023-01950-4.

## Background

In biomedical studies, sub-cohort sampling designs have been widely used to estimate biomarker-disease associations because of their cost effectiveness. Wang et al. recently developed novel two-phase sampling designs for binary outcomes [1]. Assuming that an external model is available to relate the outcome and complete covariates that are available in the first phase, the designs oversample cases and controls with worse goodness-of-fit (GOF) based on the external model and further match them on complete covariates similarly to the balanced design [2]. The GOF designs exhibit improved efficiency comparing to case–control design or a balanced case–control design for binary outcomes [1].

In cohort studies that follow subjects over time, time-to-event outcome (or survival outcome) is commonly of interest. In our motivating study from the New York University Women’s Health Study (NYUWHS), one outcome of interest is time to breast cancer diagnosis and we are interested in studying the association of hormone biomarkers and breast cancer risk in younger women [3, 4]. Although full-cohort studies provide an ideal setting to study biomarker-disease associations, the combination of large sample sizes, low incidence rates, and high costs (e.g., blood measurements) make it difficult and costly to measure the biomarkers on the entire cohort [5, 6].

Two-phase sampling designs such as nested case–control (NCC) designs [7] and case-cohort (CC) designs [8] can help overcome this limitation. Previous studies [5, 9, 10] that examined efficiency with these designs have primarily focused on comparing various inference procedures rather than from sampling design perspectives: e.g., Prentice [8], Self and Prentice [11], and Lin and Ying [6] for the un-stratified CC designs and Borgan I and II methods [12] for the stratified CC designs.

In this paper, we extend the novel GOF two-phase sampling designs proposed by Wang et al. [1] for estimating hazard ratio parameters with time-to-event data. Assuming that an external model exists to relate the survival outcome and phase I complete covariates, we propose a sampling strategy that is based on the survival probability computed from the external model as well as the follow-up time, thereby extending the GOF design to survival outcomes. For estimation and inference, we propose to use the inverse probability weighting (IPW) method to account for the sampling design.

The paper is organized as follows. In Methods section, we describe the sample designs and estimation procedures of the GOF two-phase sampling designs. Simulation of NYUWHS data section includes simulation studies evaluating the efficiency of our proposed designs based on the real dataset from NYUWHS. We conclude with Discussion section.

## Methods

### Outline and notations

Consider a cohort of $N$ subjects followed over time. Let $T=\mathrm{min}\left({T}^{*}, C\right)$ be the observed survival time (or failure time), where ${T}^{*}$ is true time-to-event (for those who develop the event) and $C$ is censoring time (for those who have not developed the event by the end of follow-up). Let $\delta =I\left({T}^{*}\le C\right)$ denote the event indicator, where the indicator function $I\left(\cdot\right)$ takes the value 1 if ${T}^{*}\le C$, and 0 otherwise. Let $X$ denote the collection of phase I covariates that are available for the entire cohort, and $Z$ denote phase II covariates (e.g. biomarkers) that can only be measured on a subset of $m \left(m\ll N\right)$. We assume that censoring time and true survival time are independent conditioning on covariates. The Cox proportional hazards (PH) regression model can be used to describe the relationship between the covariates and time-to-event outcome,

1 $$\lambda \left(t\right)={\lambda }_{0}\left(t\right){e}^{{\beta }^{T}X+{\alpha }^{T}Z},$$

where ${\lambda }_{0}\left(t\right)$ is the unknown baseline hazard function, $\beta$ and $\alpha$ are the log HR parameters for covariates $X$ and $Z$, respectively. The partial likelihood principle has been proposed to estimate the regression coefficients, $\beta$ and $\alpha$, while circumvents the estimation of infinite dimensional baseline hazard function [13, 14].

### Goodness-of-fit two-phase sampling design for time-to-event outcome

We first assume that an external working model exists and only depends on $X$, that is,

2 $${\lambda }_{e}\left(t|X\right)={\lambda }_{e0}(t){e}^{{\eta }^{T}X},$$

where ${\lambda }_{e0}(t)$, the baseline hazard function, and $\eta$, the hazard ratio parameters, are both known or can be obtained from external models. Here and in the sequel, the subscript “$e$” represents the external model. We note that such preliminary models often exist: e.g., breast cancer risk prediction models such as the Gail model [15, 16]. Note that either the complete set or a subset of $X$ can be included in the external model. We compute a GOF-based quantity for subject $i \left(i=1,\dots ,N\right)$ using the external model accounting for the length of follow-up, i.e. $D\left({T}_{i},{\delta }_{i}, {X}_{i}\right)=\left|{\delta }_{i}-{P}_{e}\left(T<{T}_{i}|{X}_{i}\right)\right|=\left|{\delta }_{i}-\left(1-{P}_{e}\left(T\ge {T}_{i}|{X}_{i}\right)\right)\right|=\left|{\delta }_{i}-1+{S}_{e}\left({T}_{i}|{X}_{i}\right)\right|,$ with survival function ${S}_{e}\left(t|X\right)=\mathrm{exp}\left(-{\int }_{0}^{t}{\lambda }_{e}\left(s|X\right)ds\right)$.

Let $R$ denote whether a subject is selected into phase II, with $R=1$ indicating selection and $R=0$ for non-selection. We propose to use the quantity $D\left({T}_{i},{\delta }_{i}, {X}_{i}\right)$ to select $m$ subjects into phase II as below, where $m={\sum }_{i=1}^{N}{R}_{i}$. Because the quantity $D$ informs the goodness-of-fit (GOF) of the external model (2), the GOF two-phase design over-samples subjects who show poor fit to the risk prediction working model as they potentially are more informative and likely show benefits from including the new phase-II biomarkers into their risk prediction. It is also desirable to achieve a prespecified case–control ratio within $m$ number of the phase II subjects as commonly done in epidemiological studies. We use the sampling probability $P\left({R}_{i}=1|{T}_{i}, {\delta }_{i}, {X}_{i}\right),$ which is $D\left({T}_{i}, {\delta }_{i}, {X}_{i}\right)$ multiplied by a constant ${c}_{1} \left({c}_{1}>0\right)$ for cases and ${c}_{0} \left({c}_{0}>0\right)$ for controls, i.e. $P\left({R}_{i}=1|{\delta }_{i}=k,{T}_{i}, {X}_{i}\right)=\mathrm{min}\left\{1, {c}_{k}D\left({T}_{i},{\delta }_{i}, {X}_{i}\right)\right\}, k=$ 0 or 1. When it is desirable to include all cases in phase II, the sampling probability for cases can be set as 1, and ${c}_{0}$ is selected to achieve the targeted number of controls by $P\left({R}_{i}=1|{\delta }_{i}=0,{T}_{i}, {X}_{i}\right)=\mathrm{min}\left\{1, {c}_{0}D\left({T}_{i},{\delta }_{i}, {X}_{i}\right)\right\}.$

Meanwhile, sub-cohort sampling designs often use a stratification on some full-cohort covariates (i.e., Phase I covariates) for various reasons: i) controlling for confounders, ii) reducing measurements error, and iii) improving efficiency of the estimates. Briefly, stratified designs first partition the cohort into different strata by confounder values (e.g., age group and race), then select random samples of sub-cohort subjects from each stratum. Our GOF sampling designs for survival outcome can also be implemented by stratifying on phase I covariates as demonstrated in Discussion section of Wang et al.[1]. When we select subjects into phase II, the balanced GOF designs allow different sampling probabilities for different strata. We term this design the balanced GOF two-phase sampling.

### Statistical inference for GOF two-phase sampling designs

Directly fitting the Cox PH model (1) to only the phase II subset selected via the GOF two-phase design can lead to the biased estimation of parameters $\beta$ and $\alpha$ because the phase II subjects are not a random representative sample of the full-cohort and are selected based on an external model using the information of outcome and phase I covariates. Thus, we propose to apply the IPW partial likelihood method for analysis, where the sampling probabilities are used as weights. Based on Eq. (1), the weighted partial likelihood function is specified as

$$PL\left(\beta,\alpha\right)={\prod\nolimits_{i=1}^m\left[\frac{w_i{\cdot e}^{\beta^TX_i+\alpha^TZ_i}}{\sum_{j=1}^mY_j\left(T_i\right)\cdot w_j\cdot e^{\beta^TX_j+\alpha^TZ_j}}\right]}^{\delta_i},$$

where ${Y}_{j}\left(t\right)=I\left({T}_{j}\ge t\right)$ is the at-risk indicator function, and ${w}_{i}=1/P\left({R}_{i}=1|{\delta }_{i}, {T}_{i}, {X}_{i}\right)$.

For the implementation, $\widehat{\beta }$, $\widehat{\alpha }$ and their standard errors can be directly estimated from standard statistical software by fitting the weighted Cox PH regression model to the phase II data (e.g., *coxph* function with the inverse of the sampling probability $P\left({R}_{i}=1|{\delta }_{i}, {T}_{i}, {X}_{i}\right)$ in the *weight* argument in the R package, “survival”) [17]. Because the weights are calculated from the external model, the standard errors of the estimates are calculated using the robust variance formula, achieved by specifying option *robust* = TRUE in the *coxph* function. Under this assumption, the variability of weight estimation is not accounted in the process of evaluating standard errors of hazard ratios of the main model. When the weights are estimated using preliminary data, other approaches such as the delta method or bootstrapping method would be considered to properly account the variability of weight estimations.

## Simulation of NYUWHS data

### Data generating process

Our simulation designs were based on the NYUWHS which consisted of 6550 women younger than 50 years of age at enrollment, where the objective was to identify risk factors for breast cancer in young women [3, 4]. As phase I covariates, we used real values of the risk factors including age at enrollment (AGE; continuous), age at menarche (AGEMEN; continuous), history of benign breast biopsy (BIOPSY; yes or no), experience of full-term pregnancy (FTP; yes or no), family history of breast cancer (REL; yes or no), and race (RACE; white or non-white). Given these covariates, we generated the time to breast cancer onset from the Eq. (1), where $X$ denoted the set of the phase I covariates and a biomarker $Z$, as a phase II covariate, was simulated as $Z=-2.15+0.05Age+\epsilon , \epsilon \sim N\left(0, 1\right)$ to yield approximately 0.2 of correlation between the $Z$ and AGE variables. The true parameter vector $\beta$ for the phase I covariates $X$ was set to be ${\left(0.028, -0.034, 0.431, -0.105, 0.541, 0.347\right)}^{T}$ based on the NYUWHS full-cohort analysis. We set $\alpha$, the coefficient for $Z$, to be 0.2 or 0.5 corresponding to a weak or strong biomarker association with disease risk, respectively. The baseline hazard function, ${\lambda }_{0}\left(t\right)$, assumed the $Weibull(k=0.929, \lambda =0.002)$. Random censoring times were independently generated from $\mathrm{min}\left(exp\left({\lambda }^{*}\right), 25\right)$, where ${\lambda }^{*}$ was set to yield a 5% or 10% event rate approximately.

### Comparisons of sub-cohort sampling designs

Under each simulation, the full-cohort analysis results were considered as the gold standard. For the GOF two-phase sampling designs, we selected phase II subjects using the sampling probability based on the GOF quantify from the external model that independently developed from a working Cox PH model ${\lambda }^{e}(t)={\lambda }_{0}^{e}(t){e}^{{\eta }^{T}X}$, using 10,000 samples bootstrapped from the full cohort data. To be comparable with the case-cohort designs, we selected all cases and used a constant ${c}_{0}$ to ensure 1-to-1 or 1-to-2 case–control ratios. We generated case-cohort data where a certain number of sub-cohort was randomly selected so that the sample sizes between our GOF two-phase sampling designs and the CC designs were almost same. Two different stratifying procedures were performed: (i) unstratified and (ii) stratified by the median of AGE variable.

We applied the standard partial likelihood method for analyzing full-cohort data and the IPW method for our GOF sampled data. As the commonly used methods for the CC designs, Prentice and Borgan I methods were applied for un-stratified CC data and stratified CC data, respectively. Because both Prentice and Borgan I methods use individual weights as the inverse of the sub-cohort selection probability, the estimation technique is essentially the same to our IPW method under the GOF two-phase designs. Therefore, we can interpret the difference in simulation results readily as the consequence of using different designs. Furthermore, we conducted the semiparametric maximum-likelihood approach (SMLE) which has been known as an efficient method among sub-cohort sampling methods using the R package, “TwoPhaseReg” [18]. The SMLE method models the conditional probability of phase II covariates given phase I covariates in the likelihood function using B-spline sieve approximation. Even though SMLE can accommodate continuous phase I covariates for analyzing two-phase data, the dimensionality of phase I covariates has to be necessarily small [18].

### Measures of model performance

The bias and standard deviation of the log hazard ratio estimates were reported as performance measures of the methods. The asymptotic standard error of the estimated log HR and the coverage probability (CP) of the 95% confidence interval (CI) were also obtained to evaluate the precision of the estimates. For comparison of the efficiency between the methods, we computed the relative efficiency as the averaged ratio of the asymptotic variances between two methods. With the setting of the large number of phase I covariates and large sample size (e.g., over 5,000 as in NYUWHS), the implementation of the SMLE method was extremely time consuming. Thus, we used random sample $N=2000$ from the full cohort of NYUWHS at each simulation, and 500 simulations were run. To investigate type I error and power of our proposed GOF two-phase designs, we additionally conducted 5,000 simulations when event rate was 5 and 10% with true $\alpha =0.2$ and $0.5$. All computations were conducted in R (version 4.0.3).

### Simulation results

The results on estimation of the biomarker’s coefficient $\alpha$ are presented in Table 1. Under the sampling designs including the full-cohort design, all estimations of $\alpha$ had negligible biases. The CPs of the 95% CIs for $\alpha$ were closed to the nominal level in all methods, indicating that the standard error estimates were accurate. Full-cohort analysis showed the highest efficiency (i.e., lowest standard deviation of the estimates) as expected. In general, the proposed GOF two-phase sampling designs showed better efficiency than the standard CC designs, and the SMLE estimation method was more efficient than IPW and weighted method for CC designs.

**Table 1:** Performance measures of the simulated biomarker coefficient $\left(\widehat{\alpha }\right)$: Bias (emp SD; CP)

Stratification	Event rate	$\alpha$	Ratio	Cox:Full cohort	IPW:Two-phase	SMLE:Two-phase	Standard:CC	SMLE:CC
Unstratified	5%	0.2	1:1	0.001 (0.098; 0.954)	0.004 (0.150; 0.936)	-0.002 (0.120; 0.944)	0.030 (0.195; 0.918)	0.005 (0.146; 0.940)
			1:2	-0.003 (0.103; 0.940)	-0.005 (0.121; 0.920)	-0.003 (0.111; 0.926)	0.010 (0.150; 0.944)	-0.002 (0.128; 0.952)
		0.5	1:1	0.008 (0.106; 0.934)	0.026 (0.163; 0.916)	0.007 (0.131; 0.946)	0.070 (0.209; 0.942)	0.013 (0.152; 0.940)
			1:2	0.003 (0.104; 0.928)	0.004 (0.124; 0.950)	0.005 (0.115; 0.948)	0.043 (0.171; 0.928)	0.002 (0.135; 0.948)
	10%	0.2	1:1	-0.003 (0.070; 0.946)	-0.009 (0.090; 0.940)	-0.005 (0.081; 0.944)	0.001 (0.123; 0.952)	-0.008 (0.099; 0.948)
			1:2	-0.001 (0.075; 0.940)	-0.007 (0.082; 0.934)	-0.001 (0.078; 0.936)	0.009 (0.096; 0.942)	0.003 (0.088; 0.946)
		0.5	1:1	0.003 (0.070; 0.940)	-0.007 (0.094; 0.954)	0.001 (0.082; 0.954)	0.042 (0.137; 0.936)	0.008 (0.100; 0.956)
			1:2	0.001 (0.074; 0.936)	-0.016 (0.081; 0.926)	0.001 (0.078; 0.940)	0.010 (0.105; 0.934)	0.000 (0.087; 0.950)
Stratified by median AGE	5%	0.2	1:1	0.001 (0.098; 0.954)	0.009 (0.146; 0.940)	0.003 (0.121; 0.944)	0.053 (0.218; 0.888)	0.008 (0.139; 0.946)
			1:2	-0.003 (0.103; 0.940)	-0.003 (0.123; 0.930)	-0.001 (0.112; 0.938)	0.024 (0.159; 0.926)	0.003 (0.129; 0.946)
		0.5	1:1	0.008 (0.106; 0.934)	0.040 (0.161; 0.936)	0.017 (0.132; 0.942)	0.143 (0.293; 0.832)	0.009 (0.157; 0.924)
			1:2	0.003 (0.104; 0.928)	0.006 (0.131; 0.936)	0.009 (0.115; 0.934)	0.069 (0.187; 0.902)	0.010 (0.131; 0.942)
	10%	0.2	1:1	-0.003 (0.070; 0.946)	-0.014 (0.088; 0.954)	-0.004 (0.080; 0.964)	0.014 (0.131; 0.934)	0.001 (0.104; 0.952)
			1:2	-0.001 (0.075; 0.940)	-0.008 (0.084; 0.930)	-0.001 (0.078; 0.944)	0.011 (0.101; 0.938)	0.005 (0.089; 0.942)
		0.5	1:1	0.003 (0.070; 0.940)	-0.004 (0.097; 0.926)	0.005 (0.085; 0.944)	0.043 (0.133; 0.948)	0.003 (0.095; 0.964)
			1:2	0.001 (0.074; 0.936)	-0.014 (0.080; 0.948)	0.001 (0.078; 0.948)	0.026 (0.114; 0.916)	0.005 (0.091; 0.934)

*Abbreviations*: *Ratio* Case and control ratio, *empSD* standard deviation of the estimates, *CP* coverage probability of the 95% CI, *Cox* Standard Cox PH model, *Full cohort* full cohort design, *IPW *IPW based Cox PH model, *Two-phase* GOF two-phase sampling design, *SMLE* semiparametric maximum-likelihood method, *Standard* Prentice method as unstratified approach and Borgan I method as stratified approach, *CC* standard case-cohort design. Note that we describe each method under each design as method:design using the abbreviations

**Fig. 1:**
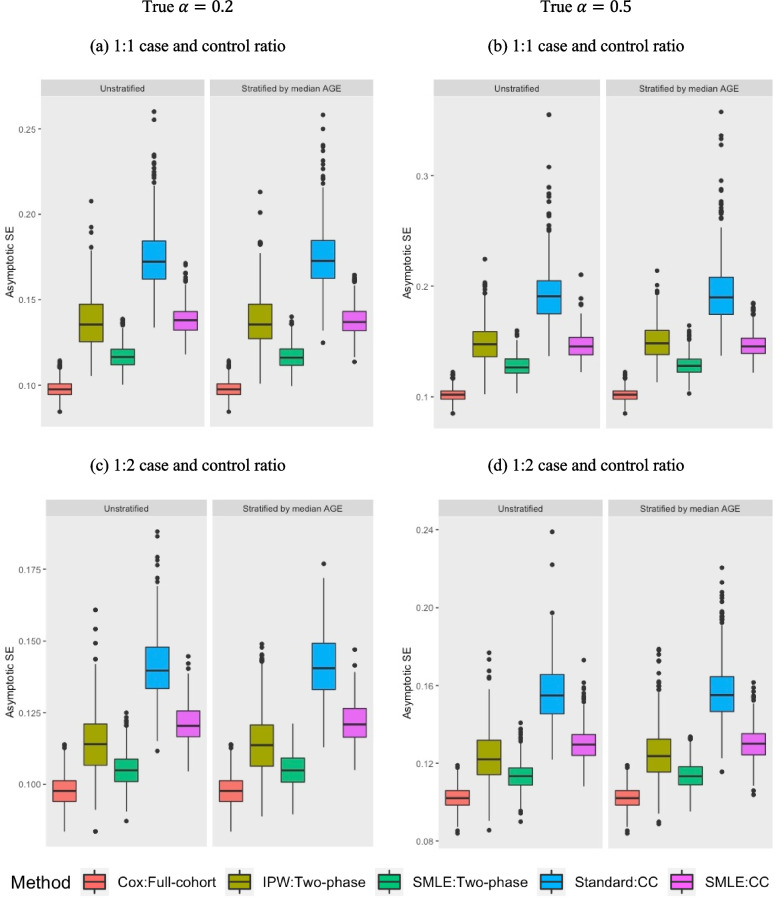
Asymptotic standard error of the estimated log HR for simulated biomarker $\left(\widehat{\alpha }\right)$ under 5% of the event rate. Abbreviations: Standard Cox PH model (Cox); full cohort design (Full cohort); IPW based Cox PH model (IPW); GOF two-phase sampling design (Two-phase); semiparametric maximum-likelihood method (SMLE); Prentice method as unstratified approach and Borgan I method as stratified approach (Standard); standard case-cohort design (CC). Note that we describe each method under each design as method:design using the abbreviations

We visualized the standard error of the estimated $\alpha$ in the case of 5% event rate (Fig. 1). The results for the 10% event rate are similar (Supplementary Fig. 1). Our proposed GOF two-phase sampling designs generally had higher efficiency than the standard CC designs. The SMLE method and the IPW under the GOF two-phase sampling design were comparably efficient. The numerical relative efficiency of the asymptotic variance of $\widehat{\alpha }$ are summarized in Table 2. In general, the proposed GOF two-phase design was more efficient compared to the standard CC design. When we compared the efficiency between each method (i.e., denominator of relative efficiency) and the SMLE method (i.e., numerator of relative efficiency) under the GOF two-phase design, the range of the relative efficiency of the IPW method was from 0.75 to 0.95 (i.e., 5–25% of efficiency loss), while standard method under the CC designs had 40–50% of additional efficiency loss. We note that the computation of SMLE can be expensive when the number of biomarker and covariates increases. Therefore, our simulations clearly demonstrated the value of novel sampling design, which can improve the efficiency of two-phase sample collection using easily implemented estimation method and is scalable to studies with large sample size and large number of biomarkers and covariates. All other phase I covariates were unbiased and showed reasonable efficiency under our proposed two-phase designs (Supplementary Tables 1 to 6).

**Table 2:** Performance measures of the simulated biomarker coefficient $\left(\widehat{\alpha }\right)$: Relative efficiency of the asymptotic variance of $\widehat{\alpha }$ under SMLE relative to each method

Stratification	Event rate	$\alpha$	Ratio	IPW:Two-phase	SMLE:Two-phase	Standard:CC	SMLE:CC
Unstratified	5%	0.2	1:1	0.744	1.000	0.533	0.720
			1:2	0.864	1.000	0.607	0.756
		0.5	1:1	0.758	1.000	0.600	0.767
			1:2	0.856	1.000	0.634	0.770
	10%	0.2	1:1	0.840	1.000	0.555	0.687
			1:2	0.920	1.000	0.685	0.765
		0.5	1:1	0.850	1.000	0.597	0.725
			1:2	0.930	1.000	0.669	0.748
Stratified by median AGE	5%	0.2	1:1	0.745	1.006	0.531	0.720
			1:2	0.869	1.003	0.602	0.754
		0.5	1:1	0.747	0.994	0.596	0.767
			1:2	0.855	1.001	0.626	0.766
	10%	0.2	1:1	0.849	1.005	0.554	0.688
			1:2	0.919	0.998	0.683	0.766
		0.5	1:1	0.848	0.994	0.594	0.722
			1:2	0.926	1.001	0.665	0.746

*Abbreviations*: *Ratio* Case and control ratio, *IPW* IPW based Cox PH model, *Two-phase* GOF two-phase sampling design, *SMLE* semiparametric maximum-likelihood method, *Standard* Prentice method as unstratified approach and Borgan I method as stratified approach, *CC* standard case-cohort design. Note that we describe each method under each design as method:design using the abbreviations. In the calculation of relative efficiency, the asymptotic variance of SMLE was numerator, while denominator was the asymptotic variance of each method

As shown in Table 3, we observed that the empirical type I error rate approached the nominal level of 0.05. The power showed that our proposed two-phase design performed increasingly well to reject the null hypothesis when the true $\alpha$ deviated from zero and with increasing event rates. Full cohort designs showed higher power than our proposed two-phase designs as expected.

**Table 3:** Type I error and power of the simulated biomarker

		Type I error	Power	
Event rate	Cohort design	$\alpha =0.0$	0.2	0.5
5%	Full cohort	0.049	0.535	0.999
	Unstratified GOF two-phase	0.055	0.320	0.973
	Stratified GOF two-phase	0.052	0.317	0.972
10%	Full cohort	0.050	0.807	1.000
	Unstratified GOF two-phase	0.052	0.581	0.999
	Stratified GOF two-phase	0.054	0.587	1.000

We considered 1:1 case and control ratio for GOF two-phase designs. The IPW method was used for GOF two-phase designs

## Discussion

Motivated by common epidemiologic time-to-event analyses, for instance, to identify risk factors of a disease in prospective cohorts, we extended the GOF two-phase sampling designs proposed by Wang et al. [1] for binary outcomes to time-to-event outcomes. We used their approach which is to oversample subjects who show poor goodness-of-fit based on an external model. We based our simulations on data from an existing study of risk factors for breast cancer in a prospective cohort, the NYUWHS. Through extensive simulations, we empirically compared our proposed method with full cohort analysis, standard weighting methods under the CC designs, and the SMLE method under both GOF two-phase sampling and CC designs. Our simulation demonstrated that inverse probability weighting methods generally showed higher efficiency in our proposed GOF two-phase sampling designs rather than the standard CC designs. Furthermore, the IPW method performed well in terms of both unbiasedness and efficiency under the GOF two-phase sampling design. Notably, balanced GOF designs achieved additional efficiency, in particular for estimating the covariates which were used for stratifying (Supplementary Tables 1 and 4). Note that this finding is consistent with the case of binary outcomes in previous study [1]. Furthermore, we also investigated the efficiency gain by the different levels of correlations between AGE variable and the simulated biomarker. Our proposed GOF two-phase designs consistently showed higher efficiency (i.e., lower than 1 of the relative efficiency) compared to standard CC designs (Supplementary Table 7).

In addition to the simulated external model used in Simulation of NYUWHS data section, we conducted simulations using the Gail model [15] with its implementation in the R package “BCRA”, which provides risk projections of invasive breast cancer according to National Cancer Institute’s Breast Cancer Risk Assessment Tool algorithm [19], to generate the GOF sampling probability. Specifically, we followed the same simulation setup of 5% event rate, true $\alpha =0.2$, and 1-to-1 case and control ratio. Using all of 6550 subjects from the NYUWHS cohort, we compared the proposed GOF two-phase designs with standard case-cohort designs. The simulation results demonstrated that the proposed GOF two-phase sampling design maintained higher efficiency (30–40% efficiency gain) than the standard CC designs (Supplementary Table 8).

Even though the SMLE promised the highest efficiency for analyzing two-phase data, it has practical limitations: i) the number of phase I covariates has to be small, especially when the covariates are continuous, and ii) the computational time heavily depends on the sample size. When the number of phase I covariates increases with the sample size, numerical cost of implementing SMLE becomes too expensive for practical use. On the other hand, the IPW method can be conveniently implemented in standard software. Furthermore, rather than randomly sampling the sub-cohort by the standard CC designs, the proposed GOF two-phase sampling design provides a new perspective to define “informative” subjects for efficient sampling, especially with respect to the potential of added values by the phase II covariates to risk characterization or prediction. By oversampling subjects with worse goodness-of-fit based on an external model, the design can include those more “informative” subjects and thus lead. to efficiency gain. This is the key idea of our proposed GOF two-phase design as in Wang et al. (2020) that the lack of fit would be suggestive of the necessity to include phase II covariate in the model to achieve better goodness-of-fit. Lastly, our proposed GOF two-phase sampling designs with the IPW method for analysis would be readily scalable in cohort studies even when the sample size is large and event rate is low.

## Supplementary Information

**Additional file 1:** **Supplementary table 1.** Simulation results for 5% event rate: Bias (SD). **Supplementary table 2.** Simulation results for 5% event rate: average SE (SD of SE). **Supplementary table 3.** Simulation results for 5% event rate: coverage probability for 95% CI. **Supplementary table 4.** Simulation results for 10% event rate: Bias (SD). **Supplementary table 5.** Simulation results for 10% event rate: average SE (SD of SE). **Supplementary table 6.** Simulation results for 10% event rate: coverage probability for 95% CI. **Supplementary table 7.** Relative efficiency of the asymptotic variance under GOF two-phase designs to standard CC designs by different level of correlations. **Supplementary table 8.** Performance measures: Bias (SD), asymptotic standard error (SD of SE) and coverage probability for 95% CI for additional simulations using the external BCRA model. **Supplementary figure 1.** Asymptotic standard error (SE) of the estimated log HR for simulated biomarker (α ^ ) from each method under each simulation setting of 10% event rate.

## Data Availability

The datasets used and R code for this study are not publicly available but are available from the corresponding author on reasonable request.
