# Supplementary material for: Goodness-of-fit two-phase sampling designs for time-to-event outcomes: a simulation study based on New York University Women’s Health Study for breast cancer
Source: BMC Med Res Methodol. 2023 May 19;23:119. doi: 10.1186/s12874-023-01950-4 (PMC10199513; doi:10.1186/s12874-023-01950-4)
Supplement: Supplementary file 1 — Additional file 1: Supplementary table 1. Simulation results for 5% event rate: Bias (SD). Supplementary table 2. Simulation results for 5% event rate: average SE (SD of SE). Supplementary table 3. Simulation results for 5% event rate: coverage probability for 95% CI. Supplementary table 4. Simulation results for 10% event rate: Bias (SD). Supplementary table 5. Simulation results for 10% event rate: average SE (SD of SE). Supplementary table 6. Simulation results for 10% event rate: coverage probability for 95% CI. Supplementary table 7. Relative efficiency of the asymptotic variance under GOF two-phase designs to standard CC designs by different level of correlations. Supplementary table 8. Performance measures: Bias (SD), asymptotic standard error (SD of SE) and coverage probability for 95% CI for additional simulations using the external BCRA model. Supplementary figure 1. Asymptotic standard error (SE) of the estimated log HR for simulated biomarker (α ^ ) from each method under each simulation setting of 10% event rate. [file 12874_2023_1950_MOESM1_ESM.docx]

**Supplementary table 1**. Simulation results for 5% event rate: Bias (SD)

| Setting | Variables | Full cohort | Two-phase design | | Balanced two-phase design | |
| --- | --- | --- | --- | --- | --- | --- |
|  |  | Gold standard | IPW | SMLE | IPW | SMLE |
| $\alpha=0.2$, 1:1 ratio | AGE | 0.001 (0.024) | -0.001 (0.035) | 0.001 (0.024) | -0.011 (0.023) | 0.001 (0.025) |
|  | AGEMEN | 0.004 (0.064) | 0.008 (0.097) | 0.004 (0.064) | 0.005 (0.095) | 0.004 (0.064) |
|  | BIOPSY | -0.014 (0.233) | 0.001 (0.357) | -0.016 (0.234) | -0.027 (0.311) | -0.016 (0.234) |
|  | FTP | 0.001 (0.195) | 0.005 (0.274) | 0.001 (0.196) | 0.010 (0.291) | 0.001 (0.196) |
|  | REL | -0.003 (0.228) | -0.029 (0.313) | -0.002 (0.229) | -0.027 (0.319) | -0.001 (0.230) |
|  | RACE | 0.002 (0.224) | -0.038 (0.319) | 0.001 (0.225) | -0.014 (0.319) | 0.002 (0.225) |
|  | BIO | 0.001 (0.098) | 0.004 (0.150) | -0.002 (0.120) | 0.009 (0.146) | 0.003 (0.121) |
| $\alpha=0.2$, 1:2 ratio | AGE | -0.001 (0.025) | -0.004 (0.030) | -0.001 (0.025) | -0.013 (0.019) | -0.001 (0.025) |
|  | AGEMEN | -0.005 (0.065) | -0.003 (0.078) | -0.005 (0.064) | -0.007 (0.075) | -0.005 (0.064) |
|  | BIOPSY | -0.019 (0.235) | -0.031 (0.285) | -0.019 (0.235) | -0.025 (0.271) | -0.019 (0.235) |
|  | FTP | 0.008 (0.198) | 0.014 (0.230) | 0.009 (0.198) | 0.019 (0.238) | 0.008 (0.198) |
|  | REL | -0.004 (0.224) | -0.038 (0.250) | -0.005 (0.224) | -0.021 (0.254) | -0.004 (0.224) |
|  | RACE | 0.010 (0.221) | -0.002 (0.270) | 0.010 (0.221) | -0.015 (0.266) | 0.009 (0.221) |
|  | BIO | -0.003 (0.103) | -0.005 (0.121) | -0.003 (0.111) | -0.003 (0.123) | -0.001 (0.112) |
| $\alpha=0.5$, 1:1 ratio | AGE | -0.001 (0.027) | 0.000 (0.039) | 0.001 (0.027) | -0.023 (0.029) | 0.000 (0.028) |
|  | AGEMEN | -0.007 (0.066) | -0.004 (0.101) | -0.007 (0.067) | -0.001 (0.106) | -0.007 (0.067) |
|  | BIOPSY | -0.001 (0.246) | -0.011 (0.350) | 0.000 (0.248) | -0.012 (0.356) | 0.001 (0.248) |
|  | FTP | 0.011 (0.225) | 0.013 (0.328) | 0.012 (0.226) | 0.017 (0.317) | 0.013 (0.226) |
|  | REL | -0.011 (0.235) | -0.047 (0.327) | -0.013 (0.241) | -0.036 (0.346) | -0.013 (0.240) |
|  | RACE | 0.005 (0.237) | -0.034 (0.353) | 0.003 (0.240) | -0.035 (0.357) | 0.005 (0.239) |
|  | BIO | 0.008 (0.106) | 0.026 (0.163) | 0.007 (0.131) | 0.040 (0.161) | 0.017 (0.132) |
| $\alpha=0.5$, 1:2 ratio | AGE | 0.000 (0.027) | -0.001 (0.032) | 0.001 (0.027) | -0.021 (0.022) | 0.001 (0.027) |
|  | AGEMEN | -0.002 (0.067) | 0.001 (0.082) | -0.002 (0.066) | 0.001 (0.081) | -0.003 (0.067) |
|  | BIOPSY | -0.012 (0.231) | -0.034 (0.286) | -0.013 (0.233) | -0.013 (0.285) | -0.014 (0.232) |
|  | FTP | 0.006 (0.212) | 0.017 (0.262) | 0.006 (0.213) | 0.011 (0.260) | 0.005 (0.212) |
|  | REL | -0.022 (0.240) | -0.049 (0.286) | -0.023 (0.241) | -0.040 (0.277) | -0.023 (0.241) |
|  | RACE | 0.002 (0.231) | -0.022 (0.289) | 0.004 (0.232) | -0.013 (0.274) | 0.002 (0.231) |
|  | BIO | 0.003 (0.104) | 0.004 (0.124) | 0.005 (0.115) | 0.006 (0.131) | 0.009 (0.115) |

Abbreviations: age at enrollment (AGE), age at menarche (AGEMEN), history of benign breast biopsy (BIOPSY), experience of full-term pregnancy (FTP), family history of breast cancer (REL), race (RACE), and simulated biomarker (BIO).

**Supplementary table 2**. Simulation results for 5% event rate: average SE (SD of SE)

| Setting | Variables | Full cohort | Two-phase design | | Balanced two-phase design | |
| --- | --- | --- | --- | --- | --- | --- |
|  |  | Gold standard | IPW | SMLE | IPW | SMLE |
| $\alpha=0.2$, 1:1 ratio | AGE | 0.025 (0.001) | 0.035 (0.003) | 0.026 (0.001) | 0.035 (0.003) | 0.026 (0.001) |
|  | AGEMEN | 0.065 (0.004) | 0.092 (0.012) | 0.065 (0.004) | 0.092 (0.011) | 0.065 (0.004) |
|  | BIOPSY | 0.236 (0.019) | 0.321 (0.027) | 0.236 (0.019) | 0.320 (0.026) | 0.236 (0.019) |
|  | FTP | 0.202 (0.010) | 0.279 (0.020) | 0.203 (0.010) | 0.279 (0.020) | 0.203 (0.010) |
|  | REL | 0.227 (0.017) | 0.306 (0.026) | 0.226 (0.016) | 0.305 (0.025) | 0.226 (0.016) |
|  | RACE | 0.221 (0.015) | 0.310 (0.024) | 0.222 (0.015) | 0.310 (0.025) | 0.222 (0.015) |
|  | BIO | 0.098 (0.005) | 0.137 (0.016) | 0.117 (0.007) | 0.137 (0.016) | 0.117 (0.007) |
| $\alpha=0.2$, 1:2 ratio | AGE | 0.025 (0.001) | 0.029 (0.003) | 0.025 (0.001) | 0.029 (0.003) | 0.025 (0.001) |
|  | AGEMEN | 0.065 (0.019) | 0.077 (0.009) | 0.065 (0.004) | 0.077 (0.009) | 0.065 (0.004) |
|  | BIOPSY | 0.236 (0.019) | 0.268 (0.021) | 0.235 (0.019) | 0.268 (0.022) | 0.235 (0.019) |
|  | FTP | 0.202 (0.010) | 0.233 (0.015) | 0.202 (0.011) | 0.234 (0.015) | 0.202 (0.011) |
|  | REL | 0.227 (0.016) | 0.256 (0.020) | 0.226 (0.016) | 0.256 (0.019) | 0.226 (0.016) |
|  | RACE | 0.221 (0.015) | 0.259 (0.019) | 0.223 (0.016) | 0.260 (0.019) | 0.223 (0.016) |
|  | BIO | 0.098 (0.005) | 0.114 (0.011) | 0.105 (0.006) | 0.114 (0.011) | 0.105 (0.006) |
| $\alpha=0.5$, 1:1 ratio | AGE | 0.026 (0.001) | 0.038 (0.004) | 0.027 (0.002) | 0.038 (0.004) | 0.027 (0.002) |
|  | AGEMEN | 0.067 (0.004) | 0.099 (0.014) | 0.068 (0.004) | 0.100 (0.013) | 0.068 (0.004) |
|  | BIOPSY | 0.243 (0.020) | 0.342 (0.032) | 0.244 (0.020) | 0.342 (0.032) | 0.244 (0.020) |
|  | FTP | 0.209 (0.011) | 0.300 (0.023) | 0.212 (0.012) | 0.301 (0.023) | 0.212 (0.012) |
|  | REL | 0.235 (0.018) | 0.330 (0.029) | 0.237 (0.018) | 0.329 (0.031) | 0.237 (0.018) |
|  | RACE | 0.228 (0.017) | 0.333 (0.028) | 0.21 (0.017) | 0.333 (0.028) | 0.231 (0.017) |
|  | BIO | 0.102 (0.006) | 0.149 (0.018) | 0.128 (0.009) | 0.150 (0.016) | 0.129 (0.009) |
| $\alpha=0.5$, 1:2 ratio | AGE | 0.026 (0.001) | 0.031 (0.003) | 0.027 (0.002) | 0.032 (0.003) | 0.027 (0.002) |
|  | AGEMEN | 0.068 (0.004) | 0.082 (0.010) | 0.068 (0.004) | 0.082 (0.010) | 0.068 (0.004) |
|  | BIOPSY | 0.243 (0.019) | 0.285 (0.024) | 0.244 (0.018) | 0.285 (0.024) | 0.244 (0.018) |
|  | FTP | 0.209 (0.011) | 0.250 (0.016) | 0.211 (0.011) | 0.250 (0.016) | 0.211 (0.011) |
|  | REL | 0.236 (0.018) | 0.276 (0.022) | 0.237 (0.018) | 0.274 (0.022) | 0.237 (0.018) |
|  | RACE | 0.228 (0.016) | 0.277 (0.020) | 0.231 (0.016) | 0.276 (0.020) | 0.231 (0.016) |
|  | BIO | 0.102 (0.006) | 0.124 (0.014) | 0.114 (0.007) | 0.124 (0.014) | 0.114 (0.007) |

**Supplementary table 3**. Simulation results for 5% event rate: coverage probability for 95% CI

| Setting | Variables | Full cohort | Two-phase design | | Balanced two-phase design | |
| --- | --- | --- | --- | --- | --- | --- |
|  |  | Gold standard | IPW | SMLE | IPW | SMLE |
| $\alpha=0.2$, 1:1 ratio | AGE | 0.962 | 0.954 | 0.958 | 0.992 | 0.964 |
|  | AGEMEN | 0.950 | 0.942 | 0.950 | 0.952 | 0.950 |
|  | BIOPSY | 0.958 | 0.924 | 0.954 | 0.956 | 0.956 |
|  | FTP | 0.962 | 0.962 | 0.956 | 0.942 | 0.956 |
|  | REL | 0.946 | 0.948 | 0.942 | 0.936 | 0.942 |
|  | RACE | 0.954 | 0.948 | 0.956 | 0.948 | 0.952 |
|  | BIO | 0.954 | 0.936 | 0.944 | 0.940 | 0.944 |
| $\alpha=0.2$, 1:2 ratio | AGE | 0.946 | 0.930 | 0.950 | 0.992 | 0.952 |
|  | AGEMEN | 0.948 | 0.942 | 0.946 | 0.960 | 0.948 |
|  | BIOPSY | 0.942 | 0.938 | 0.944 | 0.944 | 0.940 |
|  | FTP | 0.962 | 0.946 | 0.964 | 0.940 | 0.964 |
|  | REL | 0.958 | 0.956 | 0.958 | 0.960 | 0.954 |
|  | RACE | 0.958 | 0.936 | 0.958 | 0.954 | 0.960 |
|  | BIO | 0.940 | 0.920 | 0.926 | 0.930 | 0.938 |
| $\alpha=0.5$, 1:1 ratio | AGE | 0.948 | 0.940 | 0.966 | 0.964 | 0.964 |
|  | AGEMEN | 0.952 | 0.960 | 0.954 | 0.936 | 0.950 |
|  | BIOPSY | 0.952 | 0.954 | 0.952 | 0.952 | 0.950 |
|  | FTP | 0.932 | 0.930 | 0.944 | 0.940 | 0.936 |
|  | REL | 0.952 | 0.946 | 0.954 | 0.936 | 0.948 |
|  | RACE | 0.934 | 0.936 | 0.942 | 0.926 | 0.942 |
|  | BIO | 0.934 | 0.916 | 0.946 | 0.936 | 0.942 |
| $\alpha=0.5$, 1:2 ratio | AGE | 0.946 | 0.956 | 0.944 | 0.974 | 0.946 |
|  | AGEMEN | 0.950 | 0.950 | 0.958 | 0.960 | 0.956 |
|  | BIOPSY | 0.974 | 0.942 | 0.966 | 0.946 | 0.966 |
|  | FTP | 0.956 | 0.928 | 0.956 | 0.956 | 0.954 |
|  | REL | 0.950 | 0.956 | 0.952 | 0.954 | 0.956 |
|  | RACE | 0.936 | 0.944 | 0.948 | 0.958 | 0.948 |
|  | BIO | 0.928 | 0.950 | 0.948 | 0.936 | 0.934 |

**Supplementary table 4**. Simulation results for 10% event rate: Bias (SD)

| Setting | Variables | Full cohort | Two-phase design | | Balanced two-phase design | |
| --- | --- | --- | --- | --- | --- | --- |
|  |  | Gold standard | IPW | SMLE | IPW | SMLE |
| $\alpha=0.2$, 1:1 ratio | AGE | 0.000 (0.018) | -0.001 (0.022) | 0.001 (0.018) | -0.011 (0.015) | 0.001 (0.018) |
|  | AGEMEN | 0.000 (0.049) | 0.003 (0.064) | 0.000 (0.049) | 0.002 (0.063) | 0.000 (0.049) |
|  | BIOPSY | -0.007 (0.169) | -0.035 (0.213) | -0.007 (0.170) | -0.033 (0.216) | -0.007 (0.169) |
|  | FTP | -0.005 (0.133) | 0.004 (0.166) | -0.004 (0.133) | -0.001 (0.173) | -0.004 (0.133) |
|  | REL | 0.009 (0.165) | -0.016 (0.202) | 0.009 (0.165) | -0.026 (0.213) | 0.008 (0.165) |
|  | RACE | -0.011 (0.163) | -0.039 (0.202) | -0.011 (0.164) | -0.033 (0.205) | -0.011 (0.164) |
|  | BIO | -0.003 (0.070) | -0.009 (0.090) | -0.005 (0.081) | -0.014 (0.088) | -0.004 (0.080) |
| $\alpha=0.2$, 1:2 ratio | AGE | 0.000 (0.018) | -0.001 (0.020) | 0.000 (0.018) | -0.013 (0.012) | 0.000 (0.018) |
|  | AGEMEN | 0.000 (0.048) | 0.002 (0.053) | 0.000 (0.048) | 0.004 (0.051) | 0.000 (0.048) |
|  | BIOPSY | -0.007 (0.180) | -0.031 (0.188) | -0.007 (0.181) | -0.028 (0.181) | -0.008 (0.180) |
|  | FTP | -0.005 (0.145) | -0.004 (0.160) | -0.005 (0.145) | 0.002 (0.158) | -0.005 (0.145) |
|  | REL | 0.001 (0.154) | -0.029 (0.164) | 0.000 (0.155) | -0.034 (0.165) | 0.000 (0.155) |
|  | RACE | 0.001 (0.160) | -0.014 (0.176) | 0.002 (0.160) | -0.011 (0.168) | 0.001 (0.160) |
|  | BIO | -0.001 (0.075) | -0.007 (0.082) | -0.001 (0.078) | -0.008 (0.084) | -0.001 (0.078) |
| $\alpha=0.5$, 1:1 ratio | AGE | 0.001 (0.017) | 0.001 (0.023) | 0.003 (0.017) | -0.021 (0.017) | 0.003 (0.017) |
|  | AGEMEN | -0.001 (0.046) | 0.002 (0.063) | -0.001 (0.046) | 0.001 (0.064) | -0.002 (0.046) |
|  | BIOPSY | -0.017 (0.176) | -0.042 (0.226) | -0.020 (0.180) | -0.026 (0.224) | -0.020 (0.179) |
|  | FTP | -0.005 (0.149) | -0.005 (0.194) | -0.006 (0.151) | 0.004 (0.189) | -0.005 (0.150) |
|  | REL | -0.007 (0.159) | -0.038 (0.200) | -0.006 (0.162) | -0.039 (0.208) | -0.008 (0.163) |
|  | RACE | 0.011 (0.153) | -0.020 (0.212) | 0.009 (0.156) | -0.003 (0.213) | 0.011 (0.155) |
|  | BIO | 0.003 (0.070) | -0.007 (0.094) | 0.001 (0.082) | -0.004 (0.097) | 0.005 (0.085) |
| $\alpha=0.5$, 1:2 ratio | AGE | 0.002 (0.018) | 0.001 (0.019) | 0.003 (0.018) | -0.021 (0.013) | 0.003 (0.017) |
|  | AGEMEN | 0.003 (0.048) | 0.006 (0.053) | 0.003 (0.048) | 0.005 (0.052) | 0.003 (0.048) |
|  | BIOPSY | -0.009 (0.163) | -0.029 (0.182) | -0.010 (0.164) | -0.028 (0.182) | -0.009 (0.165) |
|  | FTP | 0.003 (0.141) | 0.003 (0.154) | 0.002 (0.140) | 0.006 (0.153) | 0.003 (0.140) |
|  | REL | -0.010 (0.159) | -0.041 (0.169) | -0.010 (0.159) | -0.037 (0.170) | -0.012 (0.160) |
|  | RACE | 0.009 (0.152) | -0.010 (0.175) | 0.009 (0.155) | -0.006 (0.172) | 0.010 (0.154) |
|  | BIO | 0.001 (0.074) | -0.016 (0.081) | 0.001 (0.078) | -0.014 (0.080) | 0.001 (0.078) |

**Supplementary table 5**. Simulation results for 10% event rate: average SE (SD of SE)

| Setting | Variables | Full cohort | Two-phase design | | Balanced two-phase design | |
| --- | --- | --- | --- | --- | --- | --- |
|  |  | Gold standard | IPW | SMLE | IPW | SMLE |
| $\alpha=0.2$, 1:1 ratio | AGE | 0.018 (0.001) | 0.023 (0.001) | 0.018 (0.001) | 0.023 (0.001) | 0.018 (0.001) |
|  | AGEMEN | 0.047 (0.002) | 0.061 (0.005) | 0.047 (0.002) | 0.060 (0.005) | 0.047 (0.002) |
|  | BIOPSY | 0.170 (0.010) | 0.210 (0.012) | 0.169 (0.009) | 0.210 (0.012) | 0.169 (0.009) |
|  | FTP | 0.145 (0.005) | 0.183 (0.008) | 0.146 (0.005) | 0.184 (0.008) | 0.146 (0.005) |
|  | REL | 0.164 (0.008) | 0.201 (0.010) | 0.163 (0.008) | 0.201 (0.010) | 0.163 (0.008) |
|  | RACE | 0.158 (0.007) | 0.204 (0.010) | 0.159 (0.008) | 0.204 (0.010) | 0.159 (0.008) |
|  | BIO | 0.071 (0.003) | 0.090 (0.007) | 0.082 (0.004) | 0.090 (0.007) | 0.082 (0.003) |
| $\alpha=0.2$, 1:2 ratio | AGE | 0.018 (0.001) | 0.020 (0.001) | 0.018 (0.001) | 0.020 (0.001) | 0.018 (0.001) |
|  | AGEMEN | 0.047 (0.002) | 0.051 (0.004) | 0.047 (0.002) | 0.052 (0.004) | 0.047 (0.002) |
|  | BIOPSY | 0.171 (0.011) | 0.182 (0.011) | 0.170 (0.010) | 0.182 (0.011) | 0.170 (0.010) |
|  | FTP | 0.146 (0.005) | 0.158 (0.007) | 0.146 (0.005) | 0.158 (0.007) | 0.146 (0.005) |
|  | REL | 0.164 (0.008) | 0.174 (0.009) | 0.154 (0.008) | 0.174 (0.009) | 0.164 (0.008) |
|  | RACE | 0.159 (0.008) | 0.175 (0.008) | 0.160 (0.008) | 0.175 (0.008) | 0.160 (0.008) |
|  | BIO | 0.071 (0.003) | 0.077 (0.005) | 0.074 (0.003) | 0.078 (0.005) | 0.074 (0.003) |
| $\alpha=0.5$, 1:1 ratio | AGE | 0.018 (0.001) | 0.023 (0.001) | 0.019 (0.001) | 0.023 (0.001) | 0.019 (0.001) |
|  | AGEMEN | 0.046 (0.002) | 0.061 (0.005) | 0.027 (0.002) | 0.060 (0.005) | 0.047 (0.002) |
|  | BIOPSY | 0.167 (0.009) | 0.211 (0.013) | 0.169 (0.009) | 0.210 (0.012) | 0.169 (0.009) |
|  | FTP | 0.143 (0.005) | 0.184 (0.008) | 0.145 (0.005) | 0.184 (0.008) | 0.145 (0.005) |
|  | REL | 0.162 (0.008) | 0.201 (0.011) | 0.163 (0.008) | 0.202 (0.011) | 0.163 (0.008) |
|  | RACE | 0.155 (0.007) | 0.205 (0.010) | 0.158 (0.007) | 0.205 (0.001) | 0.158 (0.007) |
|  | BIO | 0.070 (0.003) | 0.092 (0.007) | 0.084 (0.004) | 0.092 (0.007) | 0.084 (0.004) |
| $\alpha=0.5$, 1:2 ratio | AGE | 0.018 (0.001) | 0.020 (0.001) | 0.018 (0.001) | 0.020 (0.001) | 0.018 (0.001) |
|  | AGEMEN | 0.046 (0.002) | 0.051 (0.004) | 0.046 (0.002) | 0.051 (0.004) | 0.046 (0.002) |
|  | BIOPSY | 0.167 (0.009) | 0.180 (0.010) | 0.167 (0.009) | 0.180 (0.010) | 0.167 (0.009) |
|  | FTP | 0.143 (0.005) | 0.157 (0.006) | 0.144 (0.005) | 0.157 (0.006) | 0.144 (0.005) |
|  | REL | 0.162 (0.008) | 0.174 (0.009) | 0.162 (0.008) | 0.174 (0.009) | 0.162 (0.008) |
|  | RACE | 0.156 (0.007) | 0.174 (0.008) | 0.157 (0.007) | 0.174 (0.008) | 0.157 (0.007) |
|  | BIO | 0.070 (0.003) | 0.078 (0.005) | 0.075 (0.004) | 0.078 (0.005) | 0.075 (0.003) |

**Supplementary table 6**. Simulation results for 10% event rate: coverage probability for 95% CI

| Setting | Variables | Full cohort | Two-phase design | | Balanced two-phase design | |
| --- | --- | --- | --- | --- | --- | --- |
|  |  | Gold standard | IPW | SMLE | IPW | SMLE |
| $\alpha=0.2$, 1:1 ratio | AGE | 0.944 | 0.948 | 0.952 | 0.990 | 0.948 |
|  | AGEMEN | 0.940 | 0.938 | 0.934 | 0.942 | 0.936 |
|  | BIOPSY | 0.952 | 0.954 | 0.952 | 0.940 | 0.950 |
|  | FTP | 0.966 | 0.972 | 0.968 | 0.960 | 0.968 |
|  | REL | 0.950 | 0.944 | 0.950 | 0.946 | 0.958 |
|  | RACE | 0.946 | 0.956 | 0.946 | 0.952 | 0.946 |
|  | BIO | 0.946 | 0.940 | 0.944 | 0.954 | 0.964 |
| $\alpha=0.2$, 1:2 ratio | AGE | 0.944 | 0.936 | 0.950 | 0.984 | 0.958 |
|  | AGEMEN | 0.940 | 0.948 | 0.942 | 0.944 | 0.942 |
|  | BIOPSY | 0.946 | 0.958 | 0.942 | 0.964 | 0.948 |
|  | FTP | 0.954 | 0.950 | 0.956 | 0.954 | 0.960 |
|  | REL | 0.966 | 0.968 | 0.966 | 0.950 | 0.962 |
|  | RACE | 0.966 | 0.960 | 0.968 | 0.964 | 0.968 |
|  | BIO | 0.940 | 0.934 | 0.936 | 0.930 | 0.944 |
| $\alpha=0.5$, 1:1 ratio | AGE | 0.966 | 0.948 | 0.974 | 0.924 | 0.970 |
|  | AGEMEN | 0.950 | 0.934 | 0.954 | 0.926 | 0.960 |
|  | BIOPSY | 0.934 | 0.916 | 0.936 | 0.928 | 0.942 |
|  | FTP | 0.942 | 0.938 | 0.944 | 0.948 | 0.942 |
|  | REL | 0.964 | 0.950 | 0.966 | 0.940 | 0.958 |
|  | RACE | 0.950 | 0.942 | 0.956 | 0.950 | 0.950 |
|  | BIO | 0.940 | 0.954 | 0.954 | 0.926 | 0.944 |
| $\alpha=0.5$, 1:2 ratio | AGE | 0.962 | 0.950 | 0.966 | 0.920 | 0.968 |
|  | AGEMEN | 0.942 | 0.940 | 0.936 | 0.936 | 0.932 |
|  | BIOPSY | 0.964 | 0.952 | 0.958 | 0.958 | 0.958 |
|  | FTP | 0.958 | 0.960 | 0.958 | 0.966 | 0.958 |
|  | REL | 0.958 | 0.958 | 0.962 | 0.960 | 0.962 |
|  | RACE | 0.956 | 0.950 | 0.956 | 0.952 | 0.958 |
|  | BIO | 0.936 | 0.926 | 0.940 | 0.948 | 0.948 |

**Supplementary table 7.** Relative efficiency of the asymptotic variance under GOF two-phase designs to standard CC designs by different level of correlations

| $\rho$ | 0.1 | 0.3 | 0.5 | 0.7 |
| --- | --- | --- | --- | --- |
| Unstratified CC | | | | |
| AGE | 0.591 | 0.642 | 0.636 | 0.592 |
| AGEMEN | 0.597 | 0.671 | 0.665 | 0.606 |
| BIOPSY | 0.485 | 0.537 | 0.539 | 0.483 |
| FTP | 0.566 | 0.607 | 0.609 | 0.560 |
| REL | 0.470 | 0.514 | 0.510 | 0.560 |
| RACE | 0.647 | 0.685 | 0.683 | 0.468 |
| BIO | 0.589 | 0.644 | 0.629 | 0.650 |
| Stratified CC | | | | |
| AGE | 0.751 | 0.784 | 0.762 | 0.663 |
| AGEMEN | 0.616 | 0.685 | 0.665 | 0.611 |
| BIOPSY | 0.494 | 0.546 | 0.551 | 0.489 |
| FTP | 0.573 | 0.613 | 0.616 | 0.565 |
| REL | 0.472 | 0.518 | 0.521 | 0.468 |
| RACE | 0.657 | 0.699 | 0.693 | 0.655 |
| BIO | 0.608 | 0.642 | 0.644 | 0.585 |

Note: we set 5% of event rate, true $\alpha=0.2$, and 1:1 case and control ratio. Our proposed IPW method was used under the GOF two-phase designs, while Prentice and Borgan I methods were used for unstratified and stratified CC designs, respectively. In the calculation of relative efficiency, the asymptotic variance of GOF two-phase designs was numerator, while denominator was the asymptotic variance of standard CC design. The relative efficiency lass than 1 means that our GOF two-phase designs were more efficient than the standard CC designs.

**Supplementary table 8.** Performance measures: Bias (SD), asymptotic standard error (SD of SE) and coverage probability for 95% CI for additional simulations using the external BCRA model

| Designs | Full cohort | GOF two-phase sampling | Case-cohort |
| --- | --- | --- | --- |
| Methods | Cox PH | IPW | Prentice |
|  | *Bias (SD)* | | |
| AGE | 0.000 (0.014) | -0.001 (0.020) | -0.001 (0.025) |
| AGEMEN | 0.001 (0.036) | 0.002 (0.053) | -0.003 (0.067) |
| BIOPSY | 0.001 (0.130) | -0.009 (0.174) | 0.038 (0.256) |
| FTP | -0.004 (0.110) | 0.005 (0.147) | 0.009 (0.188) |
| REL | -0.007 (0.119) | -0.009 (0.163) | 0.009 (0.237) |
| RACE | 0.002 (0.122) | -0.009 (0.170) | -0.001 (0.209) |
| BIO | 0.001 (0.055) | 0.009 (0.081) | 0.011 (0.100) |
|  | *Asymptotic standard error (SD of SE)* | | |
| AGE | 0.014 (0.000) | 0.020 (0.001) | 0.024 (0.001) |
| AGEMEN | 0.036 (0.001) | 0.050 (0.003) | 0.062 (0.004) |
| BIOPSY | 0.128 (0.005) | 0.175 (0.007) | 0.239 (0.014) |
| FTP | 0.110 (0.003) | 0.154 (0.005) | 0.193 (0.008) |
| REL | 0.124 (0.004) | 0.169 (0.006) | 0.233 (0.013) |
| RACE | 0.121 (0.004) | 0.166 (0.006) | 0.198 (0.009) |
| BIO | 0.054 (0.001) | 0.076 (0.005) | 0.094 (0.006) |
|  | *Coverage probability* | | |
| AGE | 0.958 | 0.952 | 0.942 |
| AGEMEN | 0.950 | 0.944 | 0.934 |
| BIOPSY | 0.956 | 0.960 | 0.918 |
| FTP | 0.962 | 0.968 | 0.954 |
| REL | 0.948 | 0.964 | 0.958 |
| RACE | 0.952 | 0.954 | 0.942 |
| BIO | 0.950 | 0.928 | 0.936 |

Note that we assumed 5% event rate, $\alpha=0.2$, and 1:1 case and control ratio. We used the BCRA model as the external model.

**Supplementary figure 1.** Asymptotic standard error (SE) of the estimated log HR for simulated biomarker $\left( \hat{\alpha} \right)$ from each method under each simulation setting of 10% event rate

| True $\alpha=0.2$ | True $\alpha=0.5$ |
| --- | --- |
| (a) 5% event rate, 1:1 case and control ratio 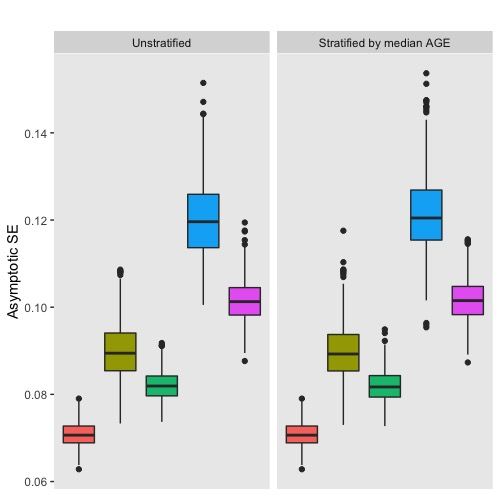 | (b) 5% event rate, 1:1 case and control ratio 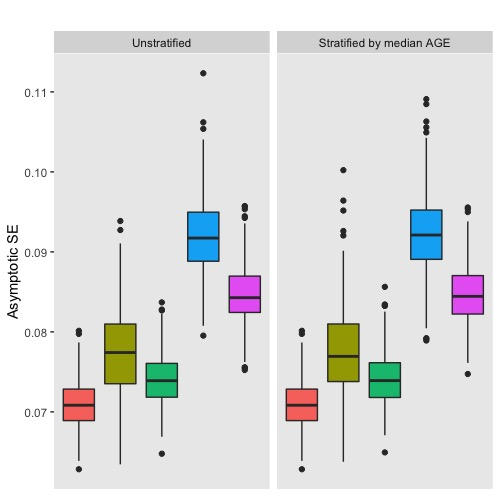 |
| (c) 5% event rate, 1:2 case and control ratio 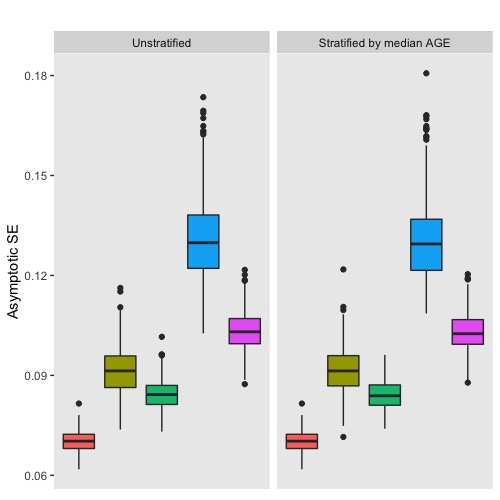 | (d) 5% event rate, 1:2 case and control ratio 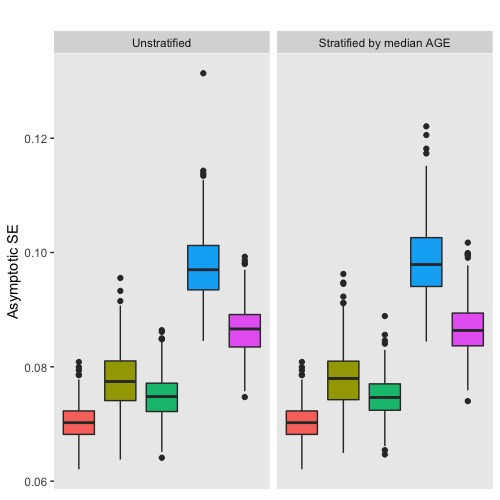 |
| 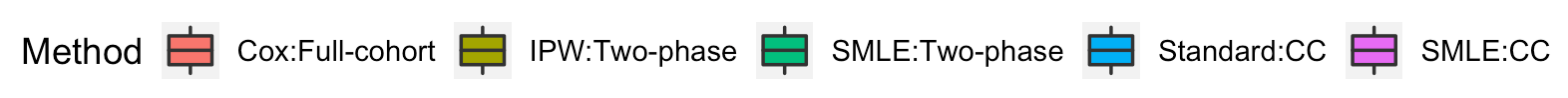 | |

Abbreviations: standard error (SE); Standard Cox PH model (Cox); full cohort design (Full cohort); IPW based Cox PH model (IPW); GOF based two-phase sampling design (Two-phase); semiparametric maximum-likelihood method (SMLE); Prentice method as unstratified approach and Borgan I method as stratified approach (Standard); standard case-cohort design (CC). Note that we describe each method under each design as method:design using the abbreviations.
